# Perioperative complications after surgical treatment in degenerative adult de novo scoliosis

**DOI:** 10.1186/s12891-017-1925-2

**Published:** 2018-01-10

**Authors:** Maciej J. K. Simon, Henry F. H. Halm, Markus Quante

**Affiliations:** 1 Department of Orthopedics, University Medical Center Hamburg-Eppendorf, Martinistrasse 52, 20246 Hamburg, Germany; 2 Department of Osteology and Biomechanics, University Medical Center Hamburg-Eppendorf, Martinistrasse 52, 20246 Hamburg, Germany; 3 Spinal Surgery Center, Schön Klink Neustadt, Am Kiebitzberg 10, 23730 Neustadt in Holstein, Germany

**Keywords:** Correction spondylodesis, Degenerative adult de novo scoliosis, Perioperative complications

## Abstract

**Background:**

Degenerative adult de novo (DAD) scoliosis appears characteristically in the sixth or seventh decade with symptoms of severe back pain and radiculopathy or spinal claudication. The aim of this study was to enhance the knowledge of perioperative complications and detect possible risk factors in this selective DAD scoliosis surgery.

**Methods:**

This retrospective study included only patients with DAD scoliosis undergone correction spondylodesis with previous failure of conservative treatment. Excluded were patients with other types of scoliosis and previous fusion surgeries. Patient epidemiological data, medical comorbidities and treatments were included. Intraoperative data and perioperative complications were documented. Analyses regarding early, late and no complications were undertaken.

**Results:**

A total of 92 patients with a mean age of 67.29 ± 7.93 years and clinical follow-up visits of minimum 12 months were included. On average, 5.26 ± 2.24 segments were fused. Early complications (e.g. wound healing defects, paresis, screw loosing) occurred in 23 patients and often required a re-operation. Cardiac arrhythmias, pacemaker and coumarin derivative therapies were associated with increased perioperative complications. The transforaminal lumbar interbody fusion technique was associated with early complications. Adjacent segment failure occurred in 36% and was the major late complication. Twenty patients did not have any complications in the minimum follow-up.

**Conclusions:**

This study analysed a selective DAD scoliosis collective and its’ surgical treatment outcomes. It identified numerous perioperative complications (adjacent segment failure, postoperative paresis and epidural hematoma) and multiple possible predisposing risk factors (e.g. operative techniques and anti-coagulation therapies). This here gained information raises awareness in preoperative patient selection and preparation. Further studies in DAD scoliosis and a risk-adjusted patient selection/preparation are needed to improve treatment quality and outcomes.

## Background

Degenerative adult de novo (DAD) scoliosis is becoming increasingly a clinically important matter [1, 2]. Most of the patients are in the sixth to seventh decade or older and suffer from severe back pain that is mostly combined with signs of spinal or foraminal stenosis. These patients have no previous history of scoliosis and develop a spinal deformity first in late adulthood [3]. The aetiology of scoliosis can be primarily degenerative, secondary to previous surgery or due to metabolic changes – in particular, osteopenia or osteoporosis – and excludes idiopathic scoliosis [1]. The prevalence varies enormously in studies and reaches levels of up to 68% in a healthy adult population [4]. The natural history of DAD scoliosis shows a progression of degenerative changes to an increasingly worse stage, leading to further deformity [1, 5, 6]. Patients can develop severe impairments, but surgery should be considered only if sufficient conservative treatment has failed and the patient is severely impaired regarding the activities of daily living.

Adult deformity surgery is technically difficult, has a high risk of complications and incompletely satisfactory outcomes [5, 7, 8]. Several intraoperative, perioperative and late-onset complications are common [9, 10]. Previous studies have identified certain surgical complications, but they contain a very heterogeneous patient collective ranging from adult idiopathic scoliosis, degenerative scoliosis, acquired, adult and iatrogenic kyphosis, and neuromuscular scoliosis, up to post-traumatic spinal deformities [10–13]. Further studies have the same problem regarding a clear risk assessment and outcome for a DAD scoliosis only collective [9, 14]. Despite previous reports, there is still a lack of information on DAD scoliosis-specific complications due to the mixture of scoliosis types in these studies. However, there seems to be a tendency towards an increased number of early complications for the group of DAD scoliosis cases.

DAD scoliosis surgery is an elective surgery and should consequently have a limited spectrum for complications. Therefore, this study premise is to collect and analyse intraoperative, perioperative and follow-up complication data of surgically treated patients exclusively with DAD scoliosis. As the next step, general and specific complications, as well as their possible risk factors for complications, were identified in this patient group in order to create a more disease surgical-specific risk pattern.

## Methods

### Diagnosis – Degenerative adult de novo (DAD) scoliosis

DAD scoliosis is a scoliosis form that develops in late adulthood. The degenerative deformation develops in the facet joints with dystrophic formation in the arthritis process or due to disc degeneration leading to frontal deviations or malrotations causing an curvature of the spine [1]. This process is mostly located in the thoracolumbar or lumbar spine [3, 15]. This form needs to be differentiated from the progressive idiopathic scoliosis. In the latter form the deformation was already present in childhood or adolescent life and progresses with increased age due to bony or degenerative changes [16, 17]. Furthermore, a distinct differentiation from secondary degenerative scoliosis with possible causes likes leg length discrepancies or vertebral fractures due to significant traumatic or metabolic changes is also fundamental [18, 19].

**Fig. 1:**
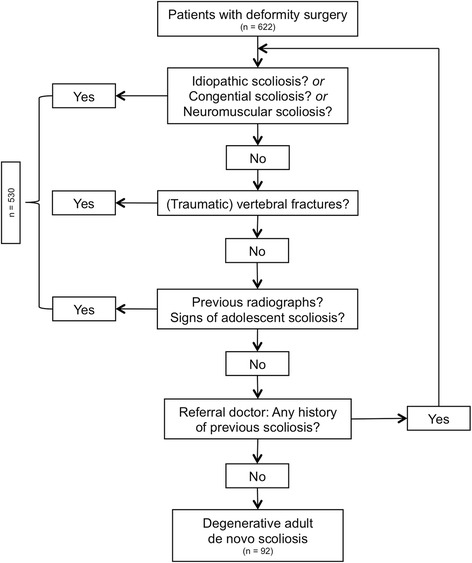
The flow chart demonstrates patients with deformity surgery (*n* = 622) from 2007 until 2009 and the exclusion process of any type of scoliosis other than of degenerative adult de novo (DAD) scoliosis (*n* = 92). An extensive patient history was undertaken. If doubts arose, previous radiographic imaging was examined. Additionally, referral doctor and/or family physician were contacted to exclude other causes for scoliosis. Exclusion occurred when idiopathic (*n* = 412), neuromuscular (*n* = 68), congenital (*n* = 33) or vertebral fractures (*n* = 17) were answered with a “Yes” or radiographic images showed idiopathic or any scoliosis deformation other than DAD scoliosis

Identification and classification of scoliosis types is part of the clinical routine. However, classification of DAD scoliosis required a comprehensive medical history work-up with extended communication of referral and/or previous doctors of the patient, evaluation and comparison of past spinal radiographs or other type of spinal images to exclude any other origin of scoliosis (Fig. 1).

Exclusion criteria included diagnosis of any type of scoliosis of other origins (e.g. idiopathic, neuromuscular, congenital), which were excluded with an extended past medical history analysis, or previous radiologic images or fusion operations.

### Study design

This retrospective study identified patients with DAD scoliosis, who underwent surgical procedures in the period from January 2007 until December 2009 with a minimum follow-up time of 12 months. During this time period, 622 deformity surgeries were performed at the authors’ institution. Most of these cases were idiopathic nature (*n* = 412) followed by degenerative (*n* = 92), neuromuscular diseases (*n* = 68), congenital and other aetiology (*n* = 33 and 17). The internal registry of the spine surgery unit documents for quality management purposes precisely all surgical procedures (including type, technique, length, etc.) after each operation and data from the routine follow-up examinations. It was established to analyse complications and perform risk analysis/stratification for each patient according to the SPINE TANGO documentation system [20]. Inclusion criteria were the diagnosis of “DAD scoliosis” with a Cobb angle of at least 10° [21], persisting symptoms, failure of conservative treatment, and the procedure of a “correction spondylodesis”.

The general symptoms were lumbago, spinal claudication and/or radicular pain. Prior to surgery, the patients had received conservative treatments (rest and avoidance of lifting heavy objects, adaption of lifestyle and antiphlogistic drugs, bracing, epidural and local facet joint injections and exercise) for a minimum of 3 to 6 months at our institution, excluding previous treatment time with the referral doctor. Only if conservative treatment failed, surgery was considered and performed after patient informed consent, including data collection, analysis and for scientific research purposes including publication followed by internal review board approval for analysis.

An ex-post analysis of prospective parameters was conducted for patients who had undergone primary correction spondylodesis in the authors’ scoliosis centre for DAD scoliosis. All procedures performed in studies involving human participants were in accordance with the ethical standards of the institutional research committee and with the 1964 Helsinki declaration and its later amendments or comparable ethical standards.

### Patient data

The patients’ epidemiological data (gender, age and body mass index (BMI)), their therapies and medical comorbidities (obesity, osteoporosis, nicotine use, diabetes, chronic obstructive pulmonary disease (COPD), hypertension, cardiac arrhythmias (CA), or pacemaker (PM) therapy) and anticoagulation therapies (such as acetylsalicylic acid or coumarin derivatives), neurological diseases (rheumatoid arthritis (RA) or steroid therapy), and whether they had previously had a non-instrumented spinal operation were preoperatively documented using a standardised procedure. The ASA (American Society of Anesthesiologists) physical status classification system was used to assess the fitness of each case before surgery.

This series represents all patients who underwent surgical treatment for DAD scoliosis between January 2007 and December 2009. None died before the minimum follow-up of 1 year, and none were lost to follow-up before that time. The mean follow-up time was 16.58 ± 13.89 months (range 12–36 months). Intraoperative data, as well as common surgical and specific spinal surgical postoperative information were analysed. Previous studies in adult deformity surgery identified several complications, however the postoperative time periods vary enormously (few days up to 12 weeks postoperatively) leading to different complication arrangements [9, 14, 22]. Due to the fact that the aim of this study was not only to include general surgical complications like wound healing, but also early problems related to the DAD scoliosis surgery (e.g. adjacent fractures, screw cut-outs), which tend to appear not immediately or are only recognized after a short observatory time and after re-gain of proper mobilization, the complication analysis led to the identification and classification of perioperative/early complications (≤ 30 days) and complications occurring >30 days after surgery.

### Intraoperative and postoperative measurements

Operation times and the number of segments fused were recorded. The operative approach (dorsal only or dorsoventral) and additional techniques, such as a vertebroplasty, cement augmentation of the screws, transforaminal or anterior lumbar interbody fusion (TLIF or ALIF), were documented and had to be individually adapted for each patient. The estimated blood loss (EBL) was recorded by the cell salvage technician based on direct analysis of the suction loss and the use of surgical sponges. Additional confirmation was performed by the anaesthesiology service. The amount of erythrocyte concentrate (red blood cell (RBC) packs), fresh frozen plasma (FFP) packs and continuous autotransfusion system (CATS) blood used was documented.

Postoperative data was divided into two sections (early (≤ 30 days) and late (> 30 days including routine follow-up examinations). This data was further divided into common surgical (e.g. wound healing defects, infections) and specific spinal spinal (e.g. epidural hematoma, dural leak) complications. Radiological controls in form of a radiograph were undertaken during the postoperative stay and in the follow-ups. If the x-rays suggested a questionable screw/rod-placement or any other doubtable lesion, or if any neurological or other unexpected symptoms occurred, a computer-tomography or MRI was executed to identify the possible cause. Re-operations were accounted for.

### Statistical analysis

Tables 1, 2, 3 and 4 report the summary statistics for all outcomes as well the summary statistics for all complications by group. Continuous variables are presented as mean ± SD, while categorical variables are presented as absolute numbers and percentages. Further, a subgroup analysis with respect to complications and all outcomes was conducted. Patients were divided into three groups, namely having experienced early (2), late (3) or no complications (4) at all. Columns 2, 3 and 4 of Tables 1, 2, 3 and 4 report the means of these subgroups and the *p*-values of their respective differences are reported in the three right-hand columns (5–7) of Tables 1, 2, 3 and 4. The *p*-values report the level of significance resulting from a *t*-test comparing the means of the subgroups. A *p*-value of ≤0.05 shows that the mean outcomes were significantly different from one another for two groups. Column 5 in Tables 1, 2, 3 and 4 reports the difference in outcomes between patients with early and late complications, column 6 between no complications and early complications, and column 7 between no and late complications.

**Table 1:** Patient epidemiological data, medical disorders and treatments

		1		2		3		4		5	6	7
		Total		Early complications (< 30 d)		Late complications (> 30 d)		No complications		*p-value* ^(3–2)^	*p-value* ^(4–2)^	*p-value* ^(4–3)^
		*n = 92*	%	*n = 23*	%	*n = 49*	%	*n = 20*	%			
	Age (years)	67.2 ± 7.9		69.1 ± 8.3		67.2 ± 7.4		65.3 ± 8.5		0.34	0.14	0.35
	BMI (kg/m^2)	26.6 ± 3.4		27.0 ± 3.2		26.8 ± 3.2		25.9 ± 4.1		0.90	0.38	0.33
Gender	Male	28	30.4	7	30.4	13	26.5	8	40.0	0.73	0.52	0.28
	Female	64	69.6	16	69.5	36	73.5	12	60.0	0.73	0.52	0.28
ASA class	1	1	1.0	0	0.0	0	0.0	1	5.0	–	0.29	0.12
	2	62	67.3	13	56.5	35	71.4	14	70.0	0.22	0.37	0.91
	3	29	31.5	10	43.4	14	28.5	5	25.0	0.22	0.21	0.77
	4	0	0.0	0	0.0	0	0.0	0	0.0	–	–	–
	Obesity	20	21.7	4	17.3	11	22.4	5	25.0	0.63	0.55	0.82
	Smoker	26	31.7	6	30.0	12	28.5	8	40.0	0.91	0.52	0.38
	Type 1 Diabetes	8	8.9	4	8.6	4	8.6	0	0.0	0.29	0.05	0.18
	Type 2 Diabetes	11	11.5	5	12.7	6	12.7	0	0.0	0.26	< 0.05	0.10
	COPD	15	16.4	6	26.0	7	14.5	2	10.0	0.25	0.18	0.62
	Osteoporosis	23	25.0	6	26.0	14	28.5	3	15.0	0.83	0.38	0.24
	Neurological Disease	4	4.3	0	0.0	4	8.1	0	0.0	0.16	–	0.19
	Hypertension	34	36.9	11	47.8	16	32.6	7	35.0	0.22	0.41	0.85
	PM	5	5.4	3	13.0	2	4.0	0	0.0	0.17	0.10	0.37
	CA	19	22.6	9	40.9	9	21.4	1	5.0	0.10	< 0.05	0.10
	RA	7	7.6	2	8.6	4	8.1	1	5.0	0.94	0.64	0.65
	Cortisone	6	6.5	2	4.0	2	4.0	2	10.0	0.43	0.89	0.35
	ASS	7	7.6	1	10.2	5	10.2	1	5.0	0.41	0.92	0.49
	Coumarin derivative	8	8.6	4	17.3	2	4.0	2	10.0	0.06	0.50	0.35

*Abbreviations: BMI* body mass index, *ASA class* American Society of Anesthesiologists, *COPD* chronic obstructive pulmonary disease, *PM* pacemaker, *CA* cardiac arrhythmias, *RA* rheumatoid arthritis, *ASS* acetylsalicylic acid, coumarin derivative – phenprocoumon. Statistical analyses regarding *p*-values were performed between the three columns/groups, i.e. early complications (2), late complications (3) and no complications (4); see superscripted numbers (significance at *p* < 0.05 or at *p* < 0.01)

**Table 2:** Intraoperative data

	1		2		3		4		5	6	7
	Total		Early complications (< 30 d)		Late complications (> 30 d)		No complications		*p-value* ^*(3–2)*^	*p-value* ^*(4–2)*^	*p-value* ^*(4–3)*^
	*n = 92*	%	*n = 23*	%	*n = 49*	%	*n = 20*	%			
Previous Spine operations -Total	27	29.3	8	34.7	15	30.6	4	20.0	0.73	0.29	0.38
Nucelotomies	8	8.6	2	8.7	4	8.2	2	10.0	0.94	0.89	0.81
Spinal canal decompressions	19	20.6	6	26	11	22.4	2	10.0	0.74	0.18	0.24
Fused segments	5.2 ± 2.2		4.8 ± 1.9		5.6 ± 2.5		4.7 ± 1.6		0.16	0.89	0.14
Dorsoventral approach	35	38	8	34.7	23	46.9	4	20.0	0.34	0.29	< 0.05
Pedicle Screw augmentation	16	17.3	6	26	6	12.2	4	20.0	0.15	0.65	0.41
Vertebroplasty	17	18.4	6	26	7	14.3	4	20.0	0.23	0.65	0.56
ALIF	39	42.3	7	30.4	27	55.1	5	25.0	0.18	0.70	0.13
TLIF	54	58.6	17	73.9	26	53	11	55.0	0.11	0.25	0.89
Operation time (min)	341.1 ± 87.6		347.1 ± 102.7		361.1 ± 77.9		296.9 ± 78.9		0.57	0.10	< 0.01
Intraoperative blood loss (ml)	1567.3 ± 1098.4		1884.0 ± 1444.4		1602.7 ± 1065.3		1178.0 ± 580.3		0.39	0.05	0.10
CATS blood (ml)	344.9 ± 355.6		577.4 ± 522.7		353.5 ± 326.7		313.2 ± 235.8		0.97	0.72	0.62
Perioperative											
RBC	3.3 ± 5.5		5.6 ± 7.2		3.2 ± 5.3		0.9 ± 1.1		0.12	< 0.05	0.06
FFP	2.5 ± 4.7		4.4 ± 6.6		2.2 ± 4.2		1.1 ± 1.5		0.10	< 0.05	0.24

*Abbreviations: ALIF and TLIF* anterior and transforaminal lumbar interbody fusion, *RBC* red blood cell packs, *FFP* fresh frozen plasma units, *CATS-blood* continuous autotransfusion system blood. Statistical analyses regarding *p*-values were performed between the three columns/groups, i.e. early complications (2), late complications (3) and no complications (4); see superscripted numbers (significance at *p* < 0.05 or at *p* < 0.01)

**Table 3:** Postoperative complications and re-operations

	1		2		3		4		5	6	7
	Total		Early complications (< 30 d)		Late complications (> 30 d)		No complications		*p-value* ^*(3–2)*^	*p-value* ^*(4–2)*^	*p-value* ^*(4–3)*^
	*n = 92*	%	*n = 23*	%	*n = 49*	%	*n = 20*	%			
Wound healing defects	17	18.4	12	52.1	5	10.2	0	0	< 0.01	< 0.01	0.14
Wound dehiscence, delayed healing	10	10.9	6	26	4	8.2	0	0	< 0.05	< 0.05	0.18
Superficial infection	7	7.6	6	26	1	2.0	0	0	< 0.01	< 0.05	0.53
Operation due to wound healing defects	13	14.1	11	47.8	2	4.1	0	0	< 0.01	< 0.01	0.37
Postoperative Paresis	13	14.1	6	26	7	14.3	0	0	0.23	< 0.05	0.08
Epidural hematoma	6	6.5	5	21.7	1	2.0	0	0	< 0.01	< 0.05	0.53
Re-Operation	6	6.5	5	21.7	1	2.0	0	0	< 0.01	< 0.05	0.53
Dural leak	7	8.7	1	5.2	6	14.6	0	0	0.30	0.31	0.07
Re-Operation	1	1.2	1	5.2	0	0	0	0	0.14	0.31	–
Adjacent segment failure Total (n)	33	35.8	12	52.1	21	42.9	0	0	0.47	< 0.01	< 0.01
Adjacent segment failure during hospital stay	8	8.7	8	34.8	0	0	0	0	< 0.01	< 0.01	–
Re-Operation	8	8.7	8	34.8	0	0	0	0	< 0.01	< 0.01	–
Adjacent segment failure later on	25	27.2	4	17.4	21	42.8	0	0	< 0.01	< 0.01	< 0.01
After how many months	9.3 ± 6.3		6.7 ± 8.0		9.85 ± 6.1		0		0.19	< 0.01	< 0.01
Re-Operation	22	23.9	3	13.0	19	38.7	0	0	< 0.01	< 0.05	< 0.01

Statistical analyses regarding *p*-values were performed between the three columns/groups, i.e. early complications (2), late complications (3) and no complications (4); see superscripted numbers (significance at *p* < 0.05 or at *p* < 0.01)

**Table 4:** Early adjacent segment failure (ASF) complications requiring surgical treatment are listed below. (Sex: F = female, M = male)

Case	Sex, Age (years)	Previous operations	Fusion length	ASF	Cause and further treatment
1	M, 62	Yes	2	Yes	Patient had persitant back pain postoperatively and paresthesia in the lower extremity. MRI showed an adjacent vertebral fracture leading to an elongation of the initial fusion of up to 4 segments and second explorative operation due to persistant paresthesia. No specific cause was identifed for this. Sensation returned to normal in the further course.
2	F, 67	No	6	Yes	Early adjacent failure was caused by an osteoporotic cut out of the upper uncemented pedicle screws. This led to an elongation of the fusion by two segements with cement-augmentation.
3	F, 74	No	5	Yes	After the inital dorsoventral fusion, an osteoporotic adjacent segment failure in the upper instrumented vertebra caused the need of a fusion extension of one more segment with pedicle screw cement augmention. Thereafter, the patient developed a wound dehiscence. This led to a further operation for wound debridement and closure.
4	M, 65	No	6	Yes	This adjacent segment failure included a pedicle fracture causing a transient paresis of the hip flexion requiring immediate screw removal and nerve exploration. There was no nerve root damage. The fusion was elongated by 2 levels. The muscle regained full function in the follow-ups.
5	F, 72	No	4	Yes	This patient had a screw misplacement as idenfied by the CT-scan. The screw was causing irritation of the nerve root and had a cut out through upper vertebral plate. This complex cause of adjacent segment failure and spinal nerve compression lead to an early revision and elongation by one segment.
6	M, 85	No	5	Yes	This patient showed during the primary operation a poor bone quality (osteoporosis) with the need to use cement augmentation of the upper instrumented vertebra. During the early mobilisation process, the patient suffered an adjacent vertebral fracture with progression needing a revision with elongation of 2 more segments with careful cement augmentation.
7	F, 67	No	6	Yes	Due to osteoporotic bone, the upper pedicle screws were cement augmented. However, the poor bone quality caused an osteoporotic fracture with nerve irritation needing a revision surgery with elongation of the fusion by 2 segments. Unfortunately thereafter, a wound healing defect caused a further superfical wound revision.
8	F, 83	No	3	Yes	Due to a collapse of the upper vertebral plate during the mobilisation process, a surgical revision with elongation of 1 segment with cement augmentation was necessary.

## Results

From a total of 622 deformity surgeries in the years of 2007 until 2009, 92 patients with a DAD scoliosis were identified and included in this retrospective analysis. After failure of conservative treatment, these patients had a primary correction spondylodesis. The average follow-up time was 16.58 ± 13.89 months (minimum 12, maximum 31 months). The mean age was 67.29 ± 7.93 years. Sixty-four patients were female (69.6%) and 28 patients were male (30.4%). The overall BMI was 26.69 ± 3.48 kg/m^2^ and 20.7% of patients were obese (BMI > 30 kg/m^2^). The physical status of the patients was within the first three ASA classes (Table 1). More than a quarter (28.3%) of the patients smoked tobacco. Nineteen patients had diabetes mellitus (8 type 1 and 11 type 2). A quarter of the entire patient group had properly diagnosed osteoporosis (T-score: ≥ −2.5). Arterial hypertension was a medical comorbidity in 34 patients (37.0%) and 19 patients (20.7%) had a CA.

Twenty-seven patients (29.3%) had a previous non-instrumented spinal operation (an open spinal nucleotomy or spinal canal decompression in 8 (8.7%) and 19 (20.7%) patients, respectively). The average number of segments fused was 5.26 ± 2.24. All cases had fusion operations in segments T12-S1 (only 21.7% had fusions extended beyond these levels – total range from T4 until S1 with iliac screws)) with an average operation time of 341.19 ± 87.66 min (Table 2). Dorsoventral spondylodesis was performed in 35 cases (38.0%), whereas TLIF was undertaken in 54 patients (58.7%). Intraoperative estimated blood loss was 1567.33 ± 1098.42 ml and the CATS blood generation averaged at 344.94 ± 355.63 ml, which led to the use of 3.30 ± 5.55 RBC and 2.55 ± 4.73 FFP units.

### Common surgical complications

In total, 17 wound healing defects were registered. Seven of these were superficial infections (Table 3). There was no correlation to diabetes mellitus or smoking. Nine postoperative electrolyte disturbances were quickly corrected and the eight urinary tract infections were uncomplicated and treated rapidly without further consequences (Table 4). There were no cases of thrombosis, pulmonary embolism or cerebrovascular insult (stroke). Two of the 92 patients died. One death within 30 days was due to postoperative disseminated intravascular coagulopathy in combination with systemic inflammatory response syndrome. The other death was due to an acute myocardial infarction several months after the operation (Table 4). The average hospital stay was 21.92 ± 10.05 days.

### Specific spinal surgical complications

Postoperative weakness/paresis occurred in 13 patients. The muscle weakness was never below stage 3 (fair function) on the Janda muscle function scale (approximately 50% strength, movement against gravity). Nine patients had muscle function of stage 4 (strength reduced, but contraction can still move the joint against reduced resistance) and four patients showed reduced muscle strength of stage 3 at hospital discharge. However, in the follow-up visits all patients had returned to full muscle strength. Epidural haematoma occurred six times and in all six cases required a re-operation. Adjacent segment failure was the major complication, which happened in a total of 33 patients. In most cases (*n* = 25), it occurred several months after initial instrumentation (9.3 ± 6.3 months) and required in 22 patients another operation.

Another frequent complication was sciatic pain, which was observed in 22 patients (23.9%). Structural diagnostics were performed in each of the cases to identify a mechanical problem (6 epidural hematomas, 1 misplacement of pedicle screws, 1 adjacent segment failure). If it was due to a mechanical cause, it was surgically repaired. If no structural cause was identified, the pain resolved itself or an intensive pain-therapy regimen had to be started (14 patients) (Table 4).

### Assessment of cases with early, late or no complications

Twenty-three patients had an early complication (≤ 30 days) following the spinal surgery. A late complication (> 30 days) occurred in a total of 49 patients. There were no significant predisposing biometric values identified regarding patient age, gender or BMI. There was no significant influence of illnesses or disorders detected regarding overall complications, but early complications tended to have a higher correlation with CA, PM and coumarin derivate therapies (Table 1).

The perioperative data show no significant differences in instrumentation length for both groups (4.82 ± 1.92 instrumented segments (early) versus 5.61 ± 2.51 (late complications)), either in the number of previous spinal surgeries or in the operating time (Table 2). A striking but non-statistically significant difference was seen in the percentage of TLIFs undertaken in the early complication group compared with the late complication group (73.9% versus 53.0%). However, the number of segments fused in these TLIF cases did not differ between these two groups. The majority of complications related to TLIF were of neurological nature. Most of these cases were classified as an early complication (6 cases of postoperative paresis, 10 cases of sciatic pain). All of these complications were transient. The postoperative paresis usually diminished within the hospital stay, whereas the sciatic pain was often prolonged but also temporary as seen in the follow-ups. Furthermore, the number of RBC and FFP units used was significantly higher in cases with early complications (Table 2).

A major late complication was adjacent segment failure (Table 3). In total, 25 cases of adjacent segment failure were observed in the follow-up visits. Twenty-two patients required surgical therapy with prolongation of the previous instrumented segments. Eight patients had an adjacent segment failure during their hospital stay (≤ 30 days) (Table 4). The causes were implant failure, screw loosening or fractured vertebral endplate, and led in all cases to a surgical revision. Sciatic pain occurred more often within 30 days postoperatively. However, intensive pain therapy was only necessary in five cases. In-hospital stay times differed significantly between the cases with early (31.65 ± 12.60 days) and late (20.16 ± 6.82 days) complications.

In comparison, a total of 20 patients did not have any complications early after the operation or later during the follow-up time of 15.80 ± 13.83 months (Table 5). These patients were slightly younger (65.3 ± 8.5 years) compared with the average age (67.29 ± 7.93 years) and had a slightly lower average BMI than the overall collective and the two complication groups (Table 1). An average of 4.75 ± 1.68 segments were fused (Table 2). Operation times were the shortest (296.90 ± 78.95 min) in comparison with the average operation time. Additionally, the estimated blood loss was the lowest at 1178 ± 580.33 ml, and thus these patients required the least amount of RBC and FFP units (Table 2).

**Table 5:** Postoperative complications with conservative treatment, in-hospital stay and follow-up times

	1		2		3		4		5	6	7
	Total		Early complications (< 30 d)		Late complications (> 30 d)		No complications		*p-value* ^*(3–2)*^	*p-value* ^*(4–2)*^	*p-value* ^*(4–3)*^
	*n = 92*	%	*n = 23*	%	*n = 49*	%	*n = 20*	%			
Electrolyte disturbances	9	9.8	2	0.09	7	0.14	0	0.00	0.51	0.19	0.08
UTI	8	8.7	3	0.15	5	0.13	0	0.00	0.79	0.07	0.10
Sciatic pain	22	23.9	12	0.52	10	0.20	0	0.00	< 0.05	< 0.01	< 0.05
Hypoesthesia	3	3.3	1	0.04	2	0.04	0	0.00	0.96	0.36	0.37
Intensive pain-therapy	14	15.2	5	0.22	9	0.18	0	0.00	0.74	< 0.05	< 0.05
Thrombosis	0	0.0	0	0.00	0	0.00	0	0.00	–	–	–
Pulmonary embolism	0	0.0	0	0.00	0	0.00	0	0.00	–	–	–
Stroke	0	0.0	0	0.00	0	0.00	0	0.00	–	–	–
Death	2	2.2	1	0.04	1	0.02	0	0.00	0.58	0.36	0.53
Hospital Stay (d)	21.9 ± 10.0		31.6 ± 12.6		20.1 ± 6.8		15.0 ± 3.2		< 0.01	< 0.01	< 0.01
Follow-up time (months)	16.4 ± 13.8		14.1 ± 10.9		17.6 ± 15.2		15.8 ± 13.8		0.32	0.67	0.63

*Abbreviations: UTI* urinary tract infection. Duration of in-hospital stay is registered in days (d) and clinical follow-up visits are documented in months. Statistical analyses regarding *p*-values were performed between the three columns/groups, i.e. early complications (2), late complications (3) and no complications (4); see superscripted numbers (significance at *p* < 0.05 or at *p* < 0.01)

## Discussion

In the aging society, patient numbers with DAD scoliosis and required surgical treatments is increasing. Specific information regarding this entity is sparse and therefore assessment of surgical complications and outcomes trying to identify risk factors is essential. The current study with a DAD scoliosis only collective demonstrates that adjacent segment failure is the main complication (35.8%). DAD scoliosis surgery bears an increased risk for various sorts of complications within 30 days after surgery as identified in 23 patients. In the further follow-up, 49 patients were documented to have a sort of late complication.

Early complications were typically wound healing dysfunctions, early adjacent segment failure, postoperative paresis or increased blood loss. Predisposing factors for these latter cases were CA, a PM or coumarin derivative therapy. In general, increased age and other co-factors (e.g. cigarette smoking, obesity or osteoporosis) are plausible contributing factors for complications following scoliosis surgery, as previously described [23]; the current results show that these can now be specifically attributed to DAD scoliosis patients as well. Factors such as CA, including PM therapy and coumarin derivative medication, were increased in patients with early complications. Generally, anticoagulation therapy management in spinal surgery is not simple [24, 25]. Poor anticoagulation management can be a cause of epidural or intraspinal haematomas, thromboembolisms or bleeding complications. However, the rate of occurrence of thromboembolisms in degenerative spinal surgeries (2.3%) is much lower than that for deformity or trauma surgery at 5.3% and 6.0%, respectively [26]. No thromboembolisms or pulmonary embolisms occurred in the current study. Postoperative haematomas may occur, but do not seem to be particularly predisposed by anticoagulative medication, as seen in the present study and a study by Cheng et al. [24].

Operative techniques, such as a TLIF, can be a risk factor for early complications, as during this process the spinal nerves are stressed, which may result in postoperative pain, sensory dysfunction or motor disabilities [27]. This is temporary in most cases, but can persist if nerves have been damaged [28]. With regard to the present results, TLIF did not seem to be a cause of major neurological complications, but should be recognised as a possible cause of minor neurological complications (transient paresis or hypoesthesia). The present results show that postoperative weakness or sensory dysfunctions resolved, in most cases, within three to 6 months after the operation. But, TLIF-technique increased operation times and blood loss, possibly leading to further postoperative complications. TLIF is not the only operation technique that may increase complication risks. Ventral surgery with support of the anterior column (ALIF) can enhance the posterior fusion stability and reconstruction of lumbar lordosis significantly [29], but the anterior approach is acquainted with an increased numbers of vascular injuries [12, 30]. However, the development of posterior fusion with TLIF techniques improved and now shows equally good results regarding fusion. But, posterior lumbar interbody fusion (PLIF) and TLIF are less effective than anterior methods in reconstructing lumbar lordosis. [31]. The selection of an operation procedure depends on the scoliosis length and severity, previous surgeries and/or bone quality and other possible confounders [32]. Therefore, each technique (e.g. ALIF) needs to be individually described to the patient carefully and explicitly explained regarding possible complications.

In contrast to our results, diabetes mellitus is regarded as a major complication factor in spinal fusion operations [33]. No significant correlation was identified in this analysed group. The results of whether insulin-dependent diabetes mellitus (IDDM) or non-insulin-dependent diabetes mellitus (NIDDM) is the more important risk factor are not completely constant but show a tendency towards IDDM [33, 34]. Cho and colleagues have shown that NIDDM was not a significant risk factor for perioperative complications or additional surgeries in spinal deformity surgery [35]. The reason for this discrepancy with diabetes being a risk factor in the literature and in the present study might be surgical invasiveness. This means that diabetes may be an influencing factor when the surgery is minor, such as in decompression operations or fusion surgeries of up to two levels, but is not of importance when fusion operations of three or more segments are performed [33–35].

The main problem in terms of early and late complications in the present study was adjacent segment failure with a total of 33 cases. Similarly, Etebar and Cahill identified in their collective that 18 of 125 patients had an adjacent segment failure problem after spinal instrumentation [36]. A majority of these were postmenopausal women. Only 22% were cigarette smokers. Most frequently, adjacent segment failure was not limited to the next segment alone. In the present study, a fair amount of patients who had adjacent segment degeneration were cigarette smoking (35.48%) or had osteoporosis (32.25%), however no significant correlation could be established. There are of course other factors that influence adjacent segment failure such as rigidity in adult spine deformities or the incorrect selection of fusion levels. These possible confounding factors were carefully considered before and during the surgery to optimize the outcome, maintain mobility/flexibility and avoidance of proximal or distal segment failure, as previous studies have analysed [37–39]. Even performance of an osteotomy confers a large risk of possible complications, but the outcome can improve significantly with such a procedure [38]. In this study of DAD scoliosis patients, 90 cases had at least three segments fused. This is often inevitable since this scoliosis type, with its degenerative nature and curvature length, make it prone to adjacent segment failure. In particular, early adjacent segment failure is of major interest, since the main reason was screw loosening or a fractured vertebral endplate, thus highlighting that the specific entity of DAD scoliosis is susceptible to early adjacent segment failure. Causes for adjacent segment failure other than misalignments in the sagittal or frontal plane are typically of a degenerative nature, based on metabolic changes which are specifically increased in this particular group of DAD scoliosis and make the adjacent segment less resilient. However, there have been no specific studies that showed a correlation between sagittal alignment and adjacent segment failure. In the current study, patients were not classified regarding their spinal balance. In future studies, the interaction between sagittal alignment and adjacent segment failure must be studied.

This study specifically focused on DAD scoliosis surgically treated patients in a retrospective manner; therefore, it has its limitations and requires careful interpretation of the results. First of all, the amount of patients included in this study is not great, but the limiting factor here was the exclusive investigation of DAD scoliosis as the sole scoliosis type, with the additional factor of no previous fusion surgeries. This might limit the case number, but leads to a clearer assessment and irreplaceable information for DAD scoliosis patients. Secondly, the follow-up times vary widely, but patients without complications and proper spinal fusion were only clinically monitored postoperatively for 1 year and thereafter should come in if problems occurred. This is not a long time to monitor patient outcomes, however, the main aspect of this retrospective study focused on perioperative and “early onset” complications, which are related to the surgical intervention. Nevertheless, patients have continuous and regular check-ups at the authors’ department, but the long-term course of the patients was not part of this study focus. Furthermore, this patient collective consists of relatively morbid and elder people that have a great tendency of rapid health situation changes. Consequently after 1 or 2 years, one monitors many phenomena/changes of aging and general degeneration that are not the direct consequences of the spondylodesis operation. Once again, the aim of this current study was to primarily identify surgical related complications of this special collective of DAD scoliosis cases and therefore focused on the time period early after surgery*.* Third, the patients in this study were not subdivided according to the specific surgical approach undertaken, with each technique having a different set of complications. This current study was rather set up to monitor general complications and their occurrences. Establishing specific risk correlations should not be done due to the heterogeneous surgical techniques used. But, to achieve the best possible outcome, we had to individually adapt the surgical techniques for each patient. Penultimate, sagittal alignment was always part of the preoperative assessment and good sagittal balance was part of the surgical aim. However, at the times of surgery it was not in the main focus, therefore we cannot draw further conclucions. This needs to be the focus of newer studies. Lastly, the small sample size prevented us from drawing conclusive evidence from the statistical mean comparisons. Only a few subgroup outcomes differed from one another, possibly due to the lack of statistical power; we may have failed to reject the null hypothesis, when we should have rejected it. Furthermore, the small sample size did not permit us to conduct in depth statistical analysis such as regression analyses. The lack of random assignment and of a control group did not permit us to interpret our results as causal.

## Conclusions

In summary, this study demonstrates that complications in DAD scoliosis surgery are not uncommon, as 25% of the patients required surgical revision within 30 days. The research exclusively limited to this very selective patient cohort is of major interest due to aging society and increased case numbers, and highlights that this elective operation has an increased risk profile. Assessing the risk factors (such as cardiac arrhythmias with anti-coagulative therapies, various operative techniques e.g. TLIF, sagittal balance) can help in planning surgical strategies, estimating risk ratios and improving outcomes. Further studies with DAD scoliosis cohorts and long-term follow-ups are required to improve the risk profiles for these patients.

## Abbreviations

ALIF: Anterior lumbar interbody fusion
ASA: American Society of Anesthesiologists
ASF: Adjacent segment failure
ASS: Acetylsalicylic acid
BMI: Body mass index
CA: Cardiac arrhythmias
CATS: Continuous autotransfusion system
COPD: Chronic obstructive pulmonary disease
d: Day(s)
DAD: Degenerative adult de novo
EBL: Estimated blood loss
F: Female
FFP: Fresh frozen plasma
IDDM: Insulin dependent diabetes mellitus
M: Male
NIDDM: Non-insulin dependent diabetes mellitus
PLIF: Posterior lumbar interbody fusion
PM: Pacemaker
RA: Rheumatoid arthritis
RBC: Red blood cell
SD: Standard deviation
TLIF: Transforaminal lumbar interbody fusion
UTI: Urinary tract infusion
